# *Abies sachalinensis* naturally growing at a sedimentary site acquires iron tolerance via detoxicants production, elemental transfer adjustment, and root endophytic *Phialocephala bamuru* producing siderophores

**DOI:** 10.1371/journal.pone.0325294

**Published:** 2025-06-17

**Authors:** Toshikatsu Haruma, Hayato Masuya, Keiko Yamaji, Yosuke Yamamoto, Naoto Nishimoto, Takahiko Arima, Shingo Tomiyama

**Affiliations:** 1 Department of Mushroom Science and Forest Microbiology, Forestry and Forest Products Research Institute, Tsukuba, Ibaraki, Japan; 2 Division of Sustainable Resources Engineering, Faculty of Engineering, Hokkaido University, Sapporo, Hokkaido, Japan; 3 Graduate School of Life and Environmental Sciences, University of Tsukuba, Tsukuba, Ibaraki, Japan.; Graphic Era Institute of Technology: Graphic Era Deemed to be University, INDIA

## Abstract

*Abies sachalinensis* naturally growing in heavy metal-rich sedimentary sites exhibits heavy metal tolerance. Although root endophytes increase heavy metal tolerance in various plants, their effects on the tolerance in conifers are not focused on. The objective of our study was to clarify the heavy metal tolerance, considering root endophytes. We measured the heavy metal concentrations in root-zone soil, leaves, stems, fine roots along with heavy metal detoxicants. We also isolated root endophytes and identified the endophyte with the highest siderophore production activity for chelating heavy metals. Results showed high Fe accumulation in fine roots, where malic acid, catechin, and condensed tannins detoxify Fe. A lower Fe/Mn ratio in leaves than roots suggests that *A. sachalinensis* could regulate Fe and Mn transfer to mitigate Fe phytotoxicity in leaves. Among the isolated root endophytes, *Phialocephala bamuru* exhibited the highest siderophore production, which could detoxify Fe in *A. sachalinensis*. These results indicated that *A. sachalinensis* have multiple Fe tolerance: Fe detoxification production and Fe/Mn ratio adjustment. Moreover, interactions with root endophytes like *Ph. bamuru* producing siderophores could increase the Fe tolerance and facilitate revegetation on soil containing heavy metal like old mine sites by conifers including *A. sachalinensis*.

## Introduction

After mine operations cease, vegetation at mine sites should be restored to promote ecological functions and protect the health of local residents. Numerous closed old mine sites exist in Japan, but they still discharge acidic mine drainage containing harmful metals at high level, which is an important problem [1]. Heavy metals are removed from the acidic mine drainage as sludge and stored at sedimentary sites. Revegetation is one of the useful and important methods to prevent heavy metals spread. Although invasive species such as fast-growing tall fescue are traditionally used for revegetation, in terms of ecological plant diversity, native plant species should be used for it [2]. In particular, sedimentary sites are located upstream, therefore, vegetation restoration is important to prevent the sediments spreading into rivers. The closed mine at our study site in Hokkaido, Japan, discharges acidic mine drainage. Neutralization and iron (Fe) removal from the drainage is still performed, and the resulting neutralized sludge is flowing into the sedimentary site used as the study site. In general, high concentrations of heavy metals in the soil disturb vegetation and inhibit plant growth [3,4]. However, several plants can be observed under heavy metal stress environments, such as sedimentary sites, indicating that plants naturally growing in sedimentary sites can adapt to heavy metal stresses [5,6]. Sakhalin fir trees (*Abies sachalinensis*) can be observed throughout the study area. *Abies sachalinensis* distribution extent throughout Hokkaido Prefecture in Japan [7] and is a dominant species, which is used for forestation, with the largest timber volume in Hokkaido Prefecture [8–10]. However, studies on the heavy metal tolerance mechanisms of *A. sachalinensis* are yet to be conducted.

As heavy metal tolerance mechanisms, plants produce detoxicants like organic acid and phenolic compounds, which reduce heavy metal toxicities [11–14]. These compounds protect plants against heavy metal toxicity by chelating heavy metals ion with highest toxicity [15–17]. Furthermore, heavy metal tolerance of plants can be increased by several microbes growing in the rhizosphere like ectomycorrhizal and arbuscular mycorrhizal fungi via suppressing the heavy metals absorption to host plants [18,19]. In addition to those microbes, recent studies have shown that root endophytes also ameliorate various environmental stresses such as salt [20,21], drought [22], and heat [23] (see also review [24]). Root endophytes are one kind of endophytes, which are defined as “fungi colonizing living plant tissue without causing any immediate, over negative effect” [25]. Among the root endophytes, specific endophytes are defined as dark septate endophytes (DSEs), which has melanized dark septa, and can infect numerous terrestrial plant species [26]. Several root endophytes including DSEs produce detoxicants called siderophores [27]. Siderophores are defined as relatively low-molecular weight compounds capable of chelating Fe [28]. Additionally, DSEs increase heavy metal tolerance of host plants by changing the heavy metal distribution [29] and producing siderophores [30]. Therefore, root endophytes including DSEs may enhance the heavy metal tolerance of *A. sachalinensis*.

The objective of our study was to clarify the heavy metal tolerance mechanisms of *A. sachalinensis* and root endophytes’ effects on the tolerance. Few researches have been conducted about heavy metal tolerance of conifer like *A. sachalinensis* with relationship of root endophytes. Firstly, we measured concentrations of heavy metals in root-zone soil and plant tissues such as leaves, stems, and fine roots of young *A. sachalinensis* trees growing in our study site during fieldwork (June–October 2022). Heavy metal detoxicants in the fine roots were determined simultaneously. We also isolated root endophytes from surface-sterilized fine root segments in August 2022 and evaluated their siderophore production activity. Lastly, we discussed the reason why *A. sachalinensis* could survive under heavy metal stress from the view point of heavy metal tolerance mechanisms considering root endophytes.

## Materials and methods

### Vegetation survey and chemical analysis of root-zone soil and *Abies sachalinensis*

Experiment permission was obtained from the respected industry and work carried out at an old mine site in Hokkaido Prefecture, Japan. No protected species were sampled in this study. Our study site (30 m × 50 m) was a sedimentary site in an old mine in the Hokkaido Prefecture, Japan. Mine wastewater, mainly including Fe, was neutralized with lime at the mine site to remove Fe as precipitation. The sedimentary site is on use and not covered with soil, showing that it is suitable field to conduct research about initial revegetation. The precipitated material was stored at our study site as soil and classified as man-made soil according to the FAO-UNESCO system [31]. The number of *A. sachalinensis* growing at our study site was counted, and their diameter at ground height was measured in August 2022. Simultaneously, root-zone soil (400 mm × 400 mm × 400 mm volume), including the root system of *A. sachalinensis*, was collected from four points at the study site. According to our vegetation survey (S1 Fig), the most abundant size individuals (1.5–2.0 cm) were collected. The soil was air-dried at 20°C for 2 weeks and passed through a sieve (<2 mm). We measured the following soil properties: pH (H_2_O), concentrations of total Al and heavy metals (Fe, Mn, Pb, and Zn), exchangeable Al, cations (Ca, Mg, K, Na), and available P and Fe, according to previously described methods (for exchangeable Al and available Fe, [32]; for the others, [33]). For pH measurement, 2 g dried soil was added into 5 mL water and mixed. After 1 h. later, the pH was determined using a pH meter (F-71; HORIBA Advanced Techno, Kyoto, Japan). Concentrations of total Al and heavy metals were determined using inductively coupled plasma optical emission spectrometry (ICP-OES; ICPE-9000, Shimadzu, Kyoto, Japan) after digestion in concentrated HNO_3_-HClO_4_ (1:4 v/v) at 140°C. Exchangeable Al was extracted from 2.0 g dried soil with 1 mol/L KCl (5 mL) for 30 min. After filtration, the soil was washed three times with 5 mL of 1 mol/L KCl. Exchangeable cations were extracted from 0.5 g dried soil with 1 mol/L CH_3_COONH_4_ (10 mL, pH 7.0) with shaking at 100 rpm for 1 h. Available P was extracted from 0.5 g dried soil with 0.5 mol/L NaHCO_3_ at pH 8.5 containing 0.1 g activated carbon with shaking at 100 rpm for 30 min. Available Fe was extracted from 2 g of dried soil with 1 mol/L CH_3_COONa at pH 4.8 and shaking at 100 rpm for 1 h. These concentrations, as well as the total concentrations of Al and heavy metals, were measured using ICP-OES. The elemental concentrations in the soils were averaged, and standard errors (SEs) were calculated.

Four *A. sachalinensis* were arbitrarily collected in June, August, and October 2022; the average tree age ± SE was 10.2 ± 1.0 years (assessed based on the number of annual rings). The collected samples were washed with deionized water to remove soil particles [34] and separated into leaves, stems, and fine roots. After the samples were dried at 80°C for 48 h, they were ground and pyrolyzed in concentrated HNO_3_ at 130°C. The concentrations of Al, Fe, Mn, Pb, and Zn in plant tissues were measured using ICP-OES. The concentrations in each tissue of the four *A. sachalinensis* plants were averaged and the SEs were calculated. The ratio between Fe and Mn concentrations (Fe/Mn ratio) is important in evaluating Fe phytotoxicity [35]; therefore, the ratio was calculated as follows:


$$\frac{Fe}{Mn}\;ratio\;=\;\frac{Fe\;concentration\;in\;each\;plant\;tissue\;(mg/kg)}{Mn\;concentration\;in\;each\;plant\;tissue\;(mg/kg)}$$

The results of the four replicates were averaged, and the SEs were calculated. The transfer factors (TFs) of Al, Fe, and Mn (ratios of plant tissue concentrations to soil concentration) for August 2022 were calculated as follows [2]:


$$Transfer\;factor\;(TF)\;=\;\frac{Metal\;element\;concentration\;in\;plant\;tissues\;(mg/kg)}{Metal\;element\;concentration\;in\;soils\;(mg/kg)}$$

The results of the four replications were averaged, and SEs were calculated.

### Analysis of organic acids and phenolic compounds in *Abies sachalinensis* fine roots

We collected four *A. sachalinensis* in August 2022 and used the fine roots for analysis of organic acids and phenolic compounds. These roots were washed with deionized water for organic acid analysis. Fine roots were cut into pieces using scissors and extracted in 80% ethanol for 1 week at 20˚C in the dark. After filtration, the fine root extract was concentrated *in vacuo* at approximately 40˚C and dissolved in 200 μL of 50% methanol. The resultant solution, equivalent to approximately 40 mg fresh weight (FW), was applied to an anion-exchange column (TOYOPAK DEAE M, Tosoh Corporation, Shunan, Japan), and the organic acids were eluted with 6 mol/L formic acid. The eluate was freeze-dried to remove the formic acid (FDU-2100; EYELA, Tokyo, Japan). The residue was dissolved in 100 μL of pyridine and 100 μL of N-methyl-N-(trimethylsilyl) trifluoroacetamide (MSTFA; Thermo Scientific, Bellefonte, PA, USA) and trimethylsilylated at 37˚C for 30 min. The organic acid concentrations were measured using gas chromatography-mass spectrometry (GC-MS) on a QP2010 instrument equipped with a GC-2010 electron-ionization mass spectrometry detector (Shimadzu) and a low-polar InertCap 5MS/Sil capillary column (30 m × 0.25 mm i.d., 0.25-μm film thickness; GL Sciences Inc., Tokyo, Japan) following the methods described in [36]. The mass spectral characteristics at *m/z* 50–1000 and the retention time of malic acid (Wako Pure Chemical Industries Ltd.)-trimethylsilyl was compared with those of the peaks in the root extracts. The absolute calibration curve of malic acid-trimethylsilyl was measured using the selected ion mode (*m/z* 73, 147, and 233) for quantification. The results of four replicates were averaged, and SEs were calculated.

For phenolic compounds analysis, washed fine roots were cut into pieces using scissors and extracted in methanol for 1 week at 20°C in the dark. After extraction, the methanol extract was filtered and concentrated *in vacuo* at approximately 40°C. The concentrate, equivalent to 25 mg FW of fine roots, was dried *in vacuo* and dissolved in 1 mL of 50% methanol. The resultant solution (10 μL) was analyzed using high-performance liquid chromatography (HPLC; Prominence UFLC series, Shimadzu) with analysis of spectral characteristics using a diode-array detector (DAD; SPD-M20A, Shimadzu) according to the method described by [37]. The spectral characteristics from 220 to 400 nm and the retention time of catechin (MP Biomedicals LLC., Santa Ana, CA, USA) were compared with the peaks of the fine root extracts. An absolute calibration curve of catechin was prepared using HPLC-DAD at 280 nm for quantification. The results of four replicates were averaged, and SEs were calculated. The molecular weight of catechin in the root extracts was measured at 280 nm using a high-performance liquid chromatography/electrospray ionization mass spectrometer (HPLC/ESI-MS; LC/MS 2020 series, Shimadzu) equipped with a UV-VIS detector (SPD-20A, Shimadzu). Nitrogen was used as the nebulizer gas (N2Supplier 24F; System Instruments, Tokyo, Japan), and MS was operated in the total ion count mode (scanning range, *m/z* 50–500). The HPLC conditions were as follows: column, Mightysil RP-18 MS (150 × 2.0 mm; Kanto, Tokyo, Japan); eluent, aq. 0.1% formic acid (solvent A) and 100% acetonitrile (solvent B); flow rate, 0.2 mL/min at 40˚C. The following gradient was used for the eluent system: 0–60 min, 100% A and 0% B to 0% A and 100% B.

The concentrations of condensed tannins were measured according to the butanol-HCl method described in [38]. Butanol reagent was prepared as follows: 0.7 g FeSO_4_·7H_2_O and 50 mL of HCl (36%) were mixed and the volume adjusted to 1000 mL with butanol. The samples (500 μL) used for HPLC-DAD analysis were added to 7 mL of butanol reagent, and reacted at 95˚C for 40 min. The absorbance of the reactants was measured at 550 nm using a UV-VIS detector (UV-1700, Shimadzu). Condensed tannins concentrations were calculated using a cyanidin chloride standard curve (Wako Pure Chemical Industries Ltd.). The concentration of condensed tannins was expressed as cyanidin chloride equivalents. The results of four replicates were averaged, and SEs were calculated.

### Microscopic observation of trypan blue stained roots and isolation of root endophytes

Four *A. sachalinensis* were collected in August 2022 and the fine roots were used for observation and isolation of root endophytes. Part of the root was used to calculate infection percentages, and the other parts were used for the isolation of root endophytes. The collected fine roots were washed with deionized water and stained with trypan blue according to a previously described method [39]. The stained roots were observed by microscopy (CX21, Olympus, Tokyo, Japan) to calculate the infection percentage of root endophytes (microsclerotia; [26,40]), arbuscular mycorrhizal fungi (*Paris*-type; [41]), and ectomycorrhizal fungi (Hartig net; [42]). The infection percentage of root length colonized was calculated according to the gridline-intersect method [43,44]. The remaining fine roots were used for root endophytes isolation. They were washed with deionized water and surface-sterilized with 70% ethanol for 1 min, 15% H_2_O_2_ for 5 min, and 70% ethanol for 1 min. They were then rinsed twice with sterile deionized water for 5 min to remove the reagents. After drying on sterile filter paper for 5 min, they were cut into 10-mm segments with a sterile scalpel, and 100 segments were randomly cut from each of the four *A. sachalinensis*. The 400 segments were incubated on 1% malt extract agar medium (1% MA) at 23°C in the dark for 2 weeks. The isolated fungi were observed by microscopy to purify. The root endophyte detection rate for each fungus was calculated using the following formula:


$$Detection\;rate\;=\;\frac{N_{d}}{N_{t}}\;\times \;100$$

where N_d_ is the number of root segments from which the fungus was detected and N_t_ is the total number of root segments used for fungal isolation (400). Morphological characteristics including colony color, growth speed, and structural features in hyphae and molecular analyses were performed to identify the most frequently isolated genera.

### Siderophore production by root endophytic fungi

The three genera of root endophytes most frequently isolated from the roots of *A. sachalinensis* were *Phialocephala* spp. (detection rate 22.3%), *Acephala* spp. (14.0%), and *Lachnum* spp. (12.3%). The siderophore production activities of these genera were determined using the chrome azurol S (CAS; TCI, Tokyo, Japan)-Fe assay. CAS-Fe agar medium was prepared according to a previously described procedure [45]. Ten isolates were randomly selected from each genus of root endophytes and grown on 1% MA for 2 weeks. Mycelial disks (5.5 mm i.d.) on the edge of each mycelium were placed on CAS-Fe agar plates (90 mm i.d.). Disks of 1% MA (5.5 mm i.d.) were used as control. The siderophore production activities were measured as below: after mycelial disks were incubated for 14-day on CAS-Fe medium at 23°C in the dark, mycelial diameters at right angles to each other were measured and averaged, and clear zone diameters were measured in the same way. The activity of each isolate was determined using the following formula:


$$Siderophore\;production\;activity\;=\;\frac{Clear\;zone\;diameter\;(mm)\;-\;Colony\;diameter\;(mm)}{Colony\;diameter\;(mm)}$$

The results of three replicates were averaged and SEs were calculated. Since *Phialocephala* spp. showed the highest activity, the activity of all 89 isolates of *Phialocephala* spp. were determined. BL211 isolate showed the highest activity and was identified using morphological characteristics and molecular analyses.

### Identification of root endophyte showing the highest siderophore production activity

DNA templates were prepared from a small piece of mycelial mass, crushed in 50 μL of sterilized water, and heated for 15 s in a microwave oven. The internal transcribed spacer (ITS) regions were amplified using primers ITS5 and ITS4 [46]. The PCR conditions were an initial denaturing step at 94˚C for 4 min; 35 cycles at 94˚C for 30 s, 52˚C for 50 s, and 72˚C for 50 s; and a final elongation at 74˚C for 6 min. The reaction mixture included 25 μL of GoTaq master mix (Promega Co., Ltd., Madison, WI, USA), 10 pmol of each primer, and 1 μL of DNA template. Amplicons were purified using a QIAquick PCR Purification Kit (Qiagen, Hilden, Germany), sequenced using a BigDye Terminator Cycle Sequencing FS Ready Reaction Kit ver. 3.1, and analyzed using an ABI3100 genetic analyzer (Applied Biosystems, Carlsbad, CA, USA). The sequences were subjected to BLAST comparisons in the National Center for Biotechnology Information database (http://www.ncbi.nlm.nih.gov/) for molecular identification [47]. The evolutionary history was inferred using the maximum likelihood method and the Kimura 2-parameter model [48]. The initial tree(s) for the heuristic search were obtained automatically by applying the neighbor-join and BioNJ algorithms to a matrix of pairwise distances estimated using the maximum composite likelihood approach and then selecting the topology with a superior log-likelihood value. A discrete gamma distribution was used to model the evolutionary rate differences among sites (five categories [+G, parameter = 0.2444]). The tree was drawn to scale and branch lengths were measured as the number of substitutions per site. The analysis included 22 nucleotide sequences. All positions with gaps or missing data were eliminated (complete deletion). In total, 434 positions were included in the final dataset. Evolutionary analyses were conducted using MEGA 11 [49].

### Statistical analysis

Statistical analyses were performed using R software for Windows (version 4.3.3) [50]. To clarify the differences due to plant tissues, differences in the concentrations and transfer factors of Al, Fe, and Mn, and Fe/Mn ratios were evaluated using one-factor ANOVA (Scheffé). Differences in the infection percentages of endophytes and mycorrhizal fungi were evaluated using Student’s *t*-test. Differences were considered statistically significant at *P* < 0.05.

## Results

### Vegetation survey of *Abies sachalinensis* and analysis of metal element concentrations in root-zone soil, leaves, stems, and roots of *A. sachalinensis*

**Fig 1:**
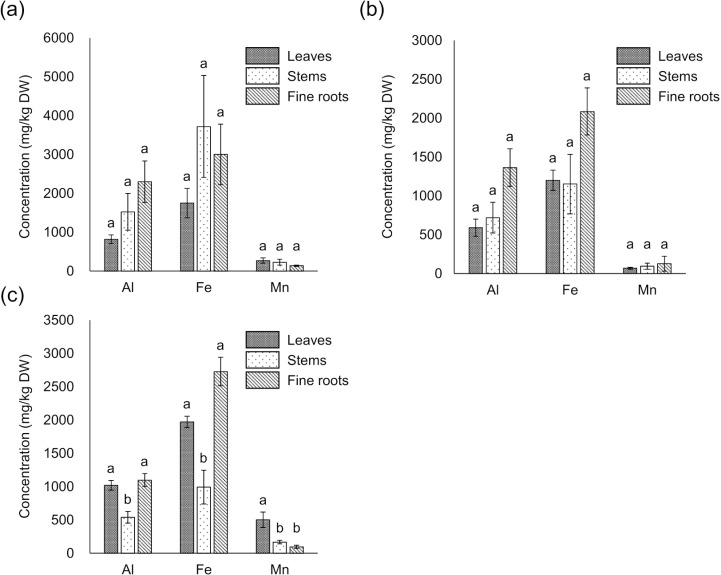
Concentrations of Al, Fe, and Mn in *A. sachalinensis* tissues. Concentrations of Al, Fe, and Mn in *A. sachalinensis* collected in (a) June, (b) August, and (c) October, 2022. Differences between treatments were evaluated using a one-factor ANOVA test (Scheffé) at *P* < 0.05 (*n* = 4). DW: dry weight; error bars represent the standard error (SE). This result shows that fine roots consistently accumulate high concentration of Fe throughout the sampling period.

**Fig 2:**
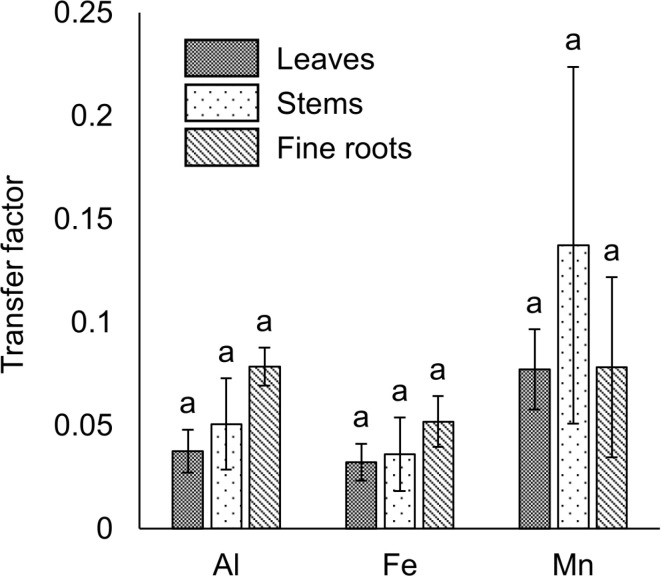
Transfer factors of Al, Fe, and Mn in *A. sachalinensis* tissues. The transfer factors for Al, Fe, and Mn in *A. sachalinensis* were determined in August 2022. Differences between treatments were evaluated using a one-factor ANOVA test (Scheffé) at *P* < 0.05 (*n* = 4). The error bars represent the standard error (SE).

**Fig 3:**
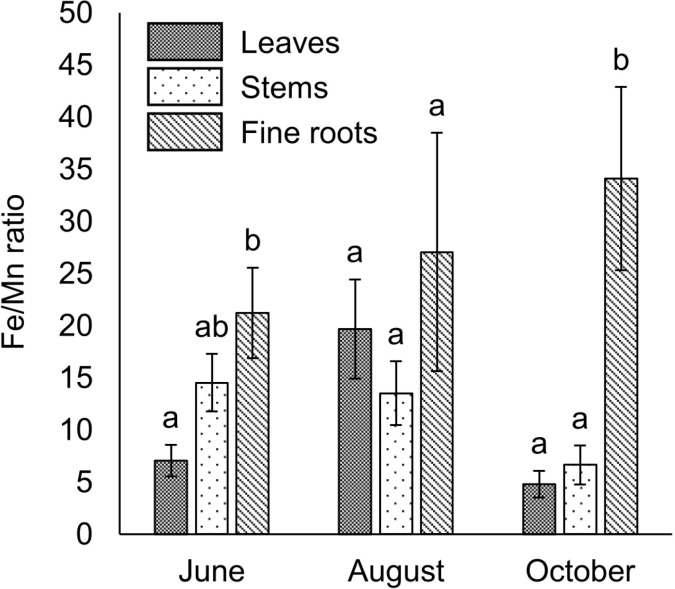
The Fe/Mn ratio in *A. sachalinensis* tissues. The Fe/Mn ratios of *A. sachalinensis* were determined in June, August, and October 2022. Differences between treatments were evaluated using a one-factor ANOVA test (Scheffé) at *P* < 0.05 (*n* = 4). The error bars represent the standard error (SE). The Fe/Mn ratio in leaves was significantly lower than roots in June and October, showing that regulated Fe and Mn transfer might mitigate Fe phytotoxicity in leaves.

The vegetation survey clarified that 117 of *A. sachalinensis* were growing at our study site and the most frequent diameter at ground height was 1.0–2.5 cm (S1 Fig). To clarify the environmental stress on *A. sachalinensis*, the root-zone soil properties were analyzed and listed in Tables 1 and 2, and the concentrations of Al, Fe, Mn, and Zn in *A. sachalinensis* tissues are listed in Fig 1. Compared with other heavy metals, much lower Pb was contained in the wastewater, from which the sediment was produced. Therefore, Pb was not detected in the root-zone soil or *A. sachalinensis* at the sedimentary site. Although Al was present at the same concentration as in the common soil (Table 1), the exchangeable concentration was low (Table 2). The root-zone soil included high concentrations of Fe, Mn, and Zn (Table 1). Among metals (Al, Fe, Mn, and Zn), the soil contained exchangeable Fe at high level despite the alkaline soil pH (Table 2), and Fe accumulated at the highest concentration (exceeding 2,000 mg/kg dry weight [DW]) in *A. sachalinensis* roots throughout the sampling period (Fig 1). The results indicate that high concentration of Fe in soil could cause stress to *A. sachalinensis*, and the tree has tolerance against Fe. The leaves and fine roots accumulated Al and Fe at higher levels than the stems (*P* < 0.05; Fig 1c), and the leaves showed significantly higher concentrations of Mn than stems and fine roots (*P* < 0.05; Fig 1c) in October. No significant differences in the TFs of Al, Fe, and Mn were found in the leaves, stems, or fine roots (Fig 2). In contrast, the Fe/Mn ratio in the fine roots was significantly higher than that in the leaves in June and October (*P* < 0.05; Fig 3).

**Table 1:** Metal elements concentrations in root-zone soil of *A. sachalinensis.*

Elements	Concentrations (mg/kg DW)
Al	18785.6 ± 5400.9
Fe	48643.0 ± 16359.8
Mn	1069.2 ± 336.5
Zn	350.0 ± 174.7

DW; dry weight. Results are expressed as mean ± standard error (SE). *n* = 4.

**Table 2:** Soil pH (H_2_O), exchangeable Al and nutrient elements, and available Fe concentrations in root-zone soil of *A. sachalinensis.*

	Values
pH (H_2_O)	8.06 ± 0.00
Exchangeable Al (mg/kg DW)	5.65 ± 3.21
Available Fe (mg/kg DW)	141.79 ± 0.56
Exchangeable Ca (mg/kg DW)	2231.57 ± 53.22
Exchangeable K (mg/kg DW)	18.14 ± 4.82
Exchangeable Mg (mg/kg DW)	182.67 ± 2.49
Exchangeable Na (mg/kg DW)	4.93 ± 2.49
Available P (mg/kg DW)	105.28 ± 6.23

DW; dry weight. Results are expressed as mean ± standard error (SE). *n* = 4.

### Concentrations of organic acids and phenolic compounds in *Abies sachalinensis* fine roots

**Fig 4:**
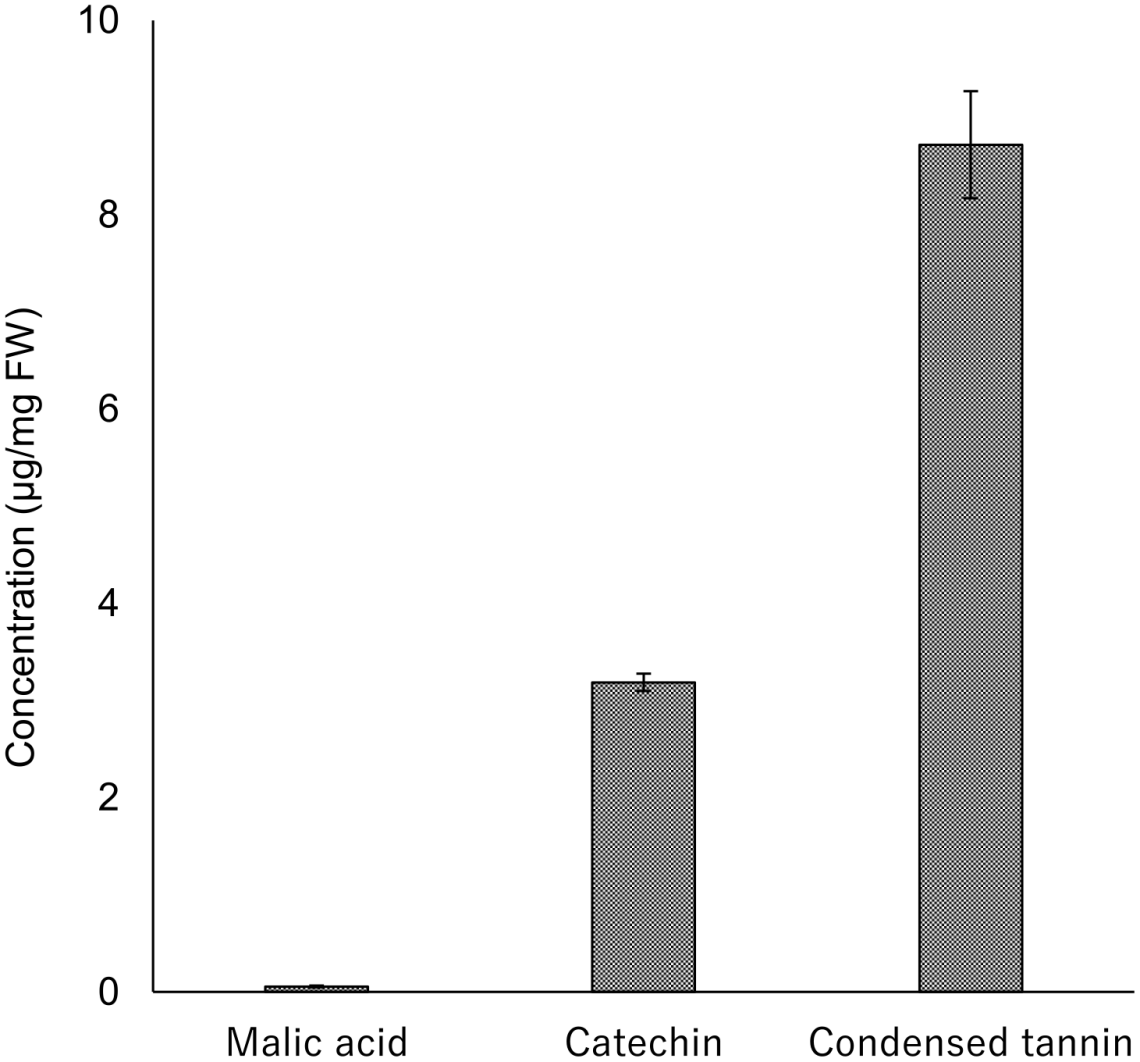
Concentrations of organic acid and phenolic compounds in *A. sachalinensis* roots. **Concentrations of malic acid, catechin, and condensed tannins. The concentration of condensed tannins was expressed as cyanidin chloride equivalents. FW: fresh weight; error bars represent the standard error (SE)**.

As one of Fe tolerance mechanisms, Fe detoxicants produced by *A. sachalinensis* were analyzed. GC/MS analysis showed that *A. sachalinensis* produced malic acid in the fine roots at a concentration of 0.06 ± 0.01 μg/mg FW (Fig 4). HPLC/ESI-MS analysis of phenolic compounds in the fine roots revealed *m/z* 291 ([M + H]^+^) and *m/z* 289 ([M-H]^-^), resulting in a molecular weight of 290. HPLC/ESI-MS and HPLC-DAD analyses clarified that *A. sachalinensis* contained catechin at a concentration of 3.19 ± 0.09 μg/mg FW (Fig 4). The butanol-HCl method revealed that the fine roots of *A. sachalinensis* contained condensed tannins at a concentration of 8.72 ± 0.55 μg/mg FW (Fig 4). Figure 4 shows that *A. sachalinensis* included malic acid, catechin and condensed tannins, which would detoxify Fe, resulting in enhancement of Fe tolerance in *A. sachalinensis.*

### Detection rate and isolation of root endophytes

**Fig 5:**
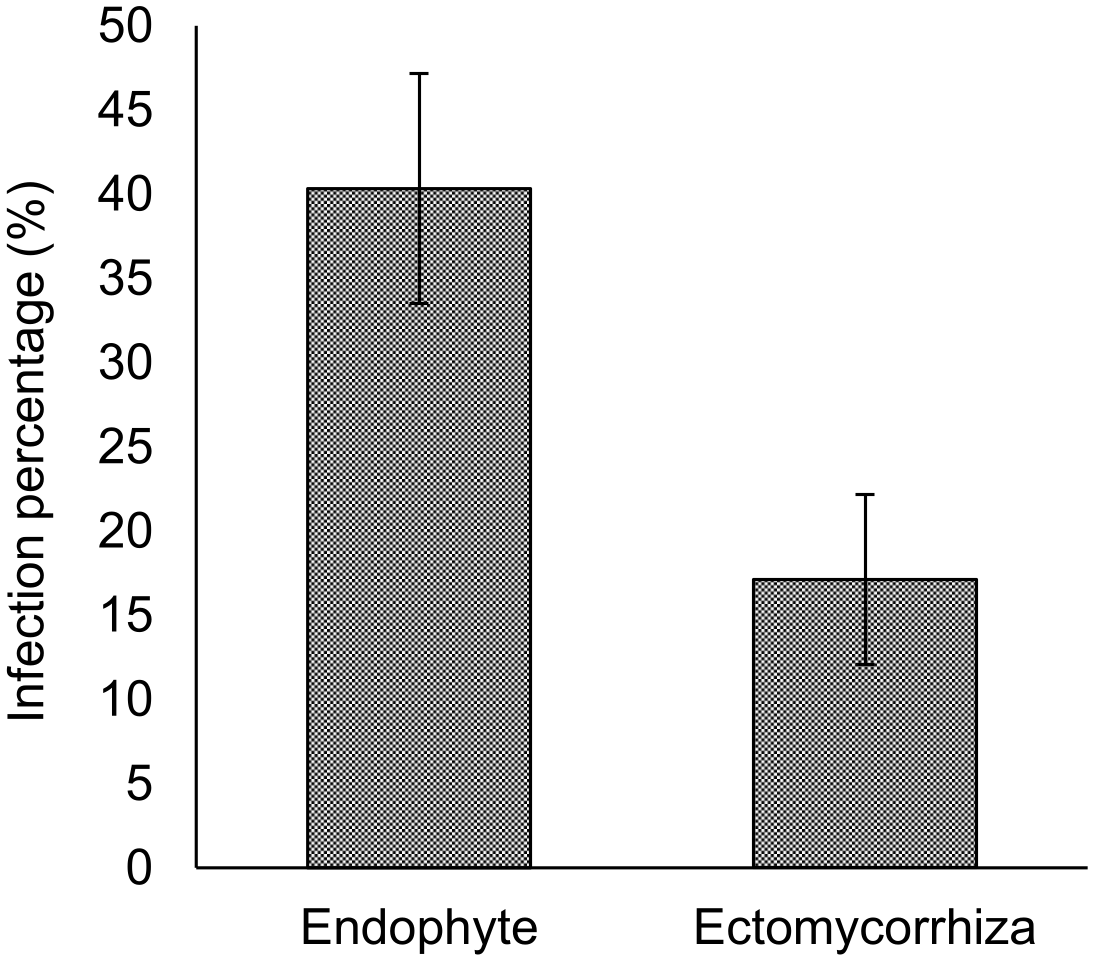
Infection percentages of endophytic fungi and ectomycorrhizal fungi. e **arResults are expressed as mean ± standard error (SE). Endophytic fungi seemed to infect at a higher percentage than ectomycorrhizal fungi, as evaluated using the Student’s *t*-test (*P* = 0.054, *n* = 4). x**.

**Fig 6:**
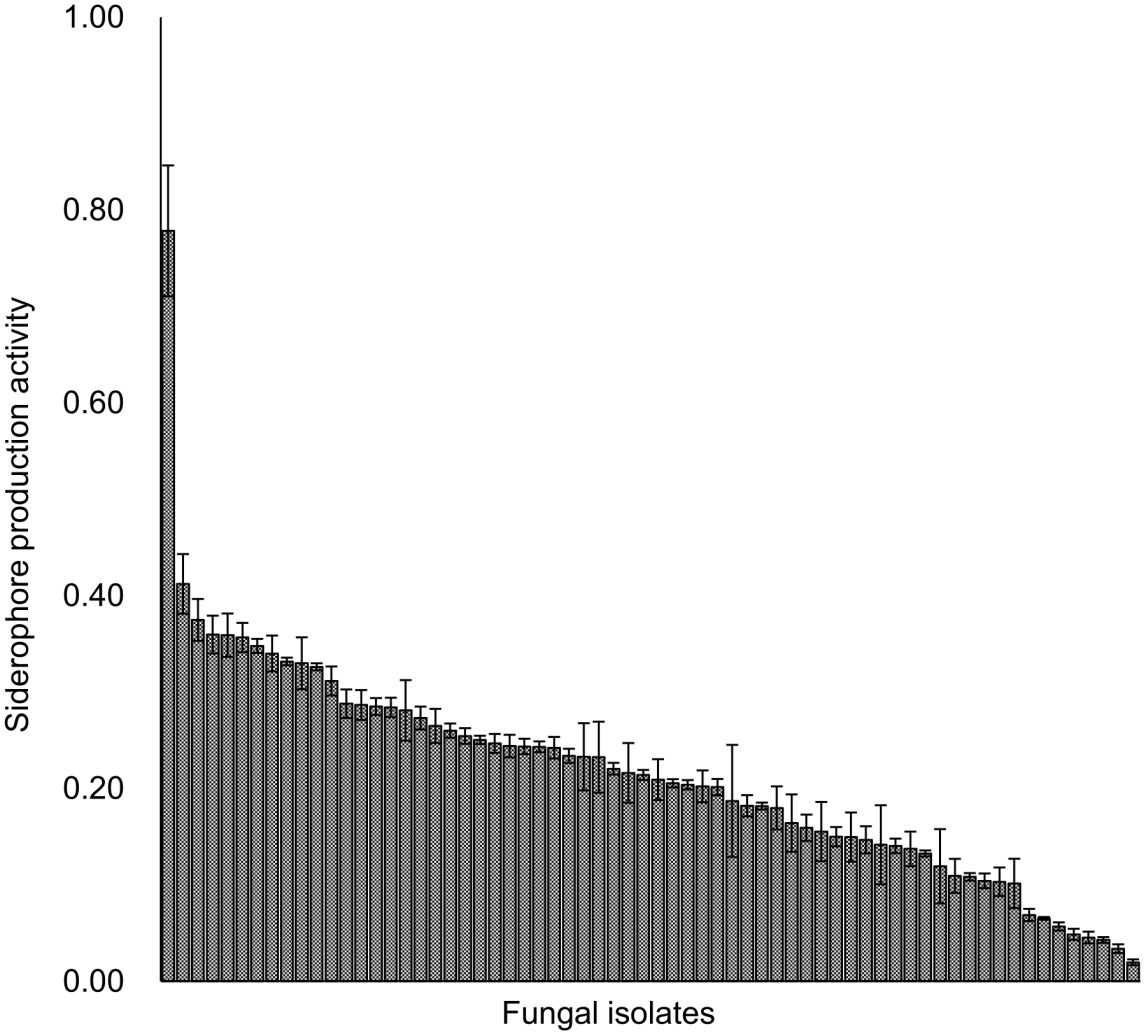
Siderophores production activities in *Phialocephala* spp. The siderophore production activity of *Phialocephala* isolates was determined. Three disks (5.5 mm i.d.) were used for each isolate to evaluate activity. BL211 isolate showed the highest activity. The error bars represent the standard error (SE).

Trypan-blue-stained fine roots observation by a microscope clarified that infection percentages by endophytes (microsclerotia) and ectomycorrhizal fungi (Hartig net) were 40.3 ± 8.6% and 17.1 ± 4.4%, respectively (Fig 5). The percentage of endophyte infections was slightly higher than that of ectomycorrhizal fungi (*P* = 0.055; Fig 5). No arbuscular mycorrhizal fungal infections were observed. Among the endophytes, the three genera frequently isolated from the fine roots of *A. sachalinensis* were *Phialocephala* (detection rate 22.3%), *Acephala* (14.0%), and *Lachnum* (12.3%). To clarify root endophytes effects on Fe tolerance in *A. sachalinensis*, their siderophore production activities were evaluated. The siderophore production activities of these fungal genera (10 isolates per genus) were determined using a CAS assay, indicating that all *Phialocephala* isolates could produce siderophores. Therefore, all 89 *Phialocephala* isolates were used to determine siderophore production activities. Among all *Phialocephala* isolates, BL211 isolate showed the highest siderophore production activity (0.78 ± 0.07; Fig 6). DNA analysis determined BL211 isolate as *Phialocephala bamuru* (S2 and S3 Fig). In this evaluation, no clear zone was showed by 1% MA disks used as control.

## Discussion

At the study site, 117 trees of *A. sachalinensis* grew naturally (S1 Fig), despite the high concentration of Fe in the soil (Table 1) compared with that in non-contaminated soil (40,000 mg/kg, [6]). The reason why there were many relatively small individuals may be that they invested carbon in their roots to acquire Fe tolerance. Generally, plants accumulate Fe at 2–700 mg/kg DW [6]. *Abies sachalinensis* accumulated more than 2,000 mg/kg DW of Fe in fine roots throughout the sampling period (Fig 1), indicating that this plant should acquire Fe tolerance in fine roots to survive at the sedimentary site. The heavy metal tolerance mechanisms of plants are mainly classified into two mechanisms: exclusion and detoxification. A high concentration of Fe in the fine roots (Fig 1) indicated that *A. sachalinensis* acquires detoxification mechanisms in the fine roots. The fine roots contained malic acid, catechin, and condensed tannins (Fig 4). These compounds possess hydroxyl groups to chelate Fe ion with highest toxicity, protecting plants against Fe toxicity [15,16,51]. Catechin also work as an antioxidant against reactive oxygen species generated by excessive Fe [15], resulting in decrease of Fe toxicity. Japanese red pine trees (*Pinus densiflora*) growing at another sedimentary site produced the same Fe detoxicants to acquire Fe tolerance [52]. Compared with their concentrations in *Pi. densiflora* [52], *A. sachalinensis* contained comparable levels of malic acid and condensed tannins and ten times the level of catechin. These results suggested that the compounds, especially catechin, allow *A. sachalinensis* to survive under Fe stress. In addition to the Fe concentration, the Fe/Mn ratio is a crucial factor for Fe phytotoxicity [35], and the ratio should be low to decrease Fe phytotoxicity. An early typical symptom of Fe phytotoxicity, causing growth inhibition, appears on the leaves as bronzing [53]. For other conifer tree, *Pi. densiflora* seedlings, high concentration of Fe cause bronzing on leaves [52]. In contrast, bronzing was not observed on the leaves of *A. sachalinensis* in our study site. The Fe/Mn ratio in the leaves was significantly lower than that in the fine roots in June and October (Fig 3). Therefore, *A. sachalinensis* might adjust Fe and Mn transfer from the fine roots to the leaves to maintain a low Fe/Mn ratio. These results indicated that *A. sachalinensis* could acquire Fe tolerance to survive at a sedimentary site via multiple mechanisms: Fe detoxicants production and regulation of Fe and Mn transfer.

The infection percentage of root endophytes seemed to be high compared with that of the ectomycorrhizal fungi (Fig 5). This result is consistent with our previous research showing that *Pi. densiflora* growing in a sedimentary site was infected by root endophytes, but not ectomycorrhizal fungi [52]. Coniferous trees such as *A. sachalinensis* are generally infected by ectomycorrhizal fungi, and even under heavy metal stress, ectomycorrhizal fungi infect host trees [54,55]. Under heavy metal stress, ectomycorrhizal fungi conferred heavy metal tolerance to their host trees; ectomycorrhizal fungus, *Cenococcum geophilum*, stimulated Masson pine trees (*Pinus massoniana*) to enhance photosynthesis, antioxidant enzyme activity, and lipid and carbohydrate synthesis. As a result, *C. geophilum* increased Cd tolerance of *Pi. massoniana* [55]. In contrast, heavy metals-rich soil disturb infection by ectomycorrhizal fungi [56,57]. For instance, compared with non-contaminated forest soil, tailing soil containing Pb, Zn, and Cu decreased the ectomycorrhizal colonization rate of *Pi. massoniana* roots from 61.4 ± 29.0% to 25.9 ± 21.0% [57]. Infection by ectomycorrhizal fungi is affected by the amount of organic carbon in the soil [58], poor nutrient properties, and heavy metal concentrations [59] (see also review [18]). Moreover, high concentrations of heavy metals in soil inhibit the growth of living fine roots, which are a source of forming ectomycorrhizal symbiotic relationships, resulting in reduced biomass of ectomycorrhizal fungi [54]. Our study site has been used as a sedimentary site, and sediment emitted from a mine is still added; therefore, the root-zone soil contained low concentrations of nutrients (Table 2) and high concentrations of heavy metals (Table 1), which would inhibit ectomycorrhizal fungal infection to *A. sachalinensis*. Therefore, at the sedimentary site, root endophytes could replace ectomycorrhizal fungi and play important roles for *A. sachalinensis*. These results indicated that root endophytes could mainly protect *A. sachalinensis* against heavy metals and enhance the establishment of *A. sachalinensis* under severe environmental stress when it is difficult for ectomycorrhizal fungi to infect host plants.

*Phialocephala* spp., most frequently isolated in this study, has dark melanized septa and are classified into DSEs according to [24]. Melanized hyphae can bind heavy metals [60]; therefore, *Phialocephala* spp. isolated in our study could contribute to reducing Fe toxicity in *A. sachalinensis* via adsorbing Fe into their hyphae in plant cells. *Phialocephala* isolate (BL211) showed the highest siderophore production activity (Fig 6), and DNA analysis identified it as *Ph. bamuru*. This result indicated that *Ph. bamuru* would produce siderophores to protect *A. sachalinensis* against Fe stress. In particular, under heavy metal-rich environment, where other symbiotic microbes like arbuscular mycorrhizal and ectomycorrhizal fungi could not support *A. sachalinensis*, *Ph. bamuru* should be an important survival factor for *A. sachalinensis*. Among *Phialocephala* species, *Phialocephala fortinii* has been well investigated as a DSE [26], and the effects of *Ph. fortinii* on plant growth under heavy metal stress have been evaluated. For example, *Ph. fortinii* promotes the growth of pepperbush trees (*Clethra barbinervis*) via promoting the absorption of nutrient element under heavy metal stress [61]. Similarly, *Ph. fortinii* stimulates the growth of silver grass (*Miscanthus sinensis*) under Al stress by producing a phytohormone (indole-3-acetic acid)-like compound [62]. For heavy metal detoxicants production, *Ph. fortinii* produces hydroxamate siderophores [27]. Compared with *Ph. fortinii*, few studies were conducted on *Ph. bamuru* and its functions in plants remain unclear. For example, *Ph. bamuru* causes severe emerging diseases in Bermuda grass (*Cynodon dactylon*) and kikuyu grass (*Pennisetum clandestinum*) in Australia [63]. In contrast, *Ph. bamuru* protected Scotch pine (*Pinus sylvestris*) from the phytopathogenic fungus, *Rhizoctonia solani* [64]. Endophytes change their roles in host plants depending on the environment and host plants status [65,66]. In the present study, *Ph. bamuru* would increase Fe tolerance in the host plant via producing siderophores (Fig 6), thereby facilitating the establishment of *A. sachalinensis* under heavy metal stress. These results suggest that without sowing alien species, using native interactions like planting *A. sachalinensis* seedlings inoculated by *Ph. bamuru* is ecologically beneficial for revegetation on old mine sites. Henceforth, producing *A. sachalinensis* seedlings inoculated by *Ph. bamuru* might promote revegetation on heavy metal environment.

## Conclusion

*Abies sachalinensis* is a dominant forest component tree, which naturally grows throughout Hokkaido Prefecture; therefore, it should be an ecologically useful tree species for revegetation at heavy metal-rich environment. In addition to heavy metal tolerance in *A. sachalinensis*, native root endophytes should also adapt the trees to severe environment. However, few researches focus on the heavy metal tolerance in *A. sachalinensis* and interaction with root endophytes increasing the tolerance. We discussed the Fe tolerance mechanisms of *A. sachalinensis* to survive Fe contaminated soil, such as a sedimentary site considering root endophytes. Fe detoxicants, such as malic acid, catechin, and condensed tannins could contribute on Fe tolerance in *A. sachalinensis* to adapt to the severe environment. Moreover, Fe and Mn transfer from fine roots to leaves might be adjusted to keep the Fe/Mn ratio in the leaves low, alleviating Fe toxicity in the leaves. These results suggested that Fe detoxicants and low Fe/Mn ratio could allow *A. sachalinensis* to survive under Fe stress environments. As a root endophyte, *Ph. bamuru* was isolated from the fine roots of *A. sachalinensis* and showed siderophore production activity; therefore, *Ph. bamuru* would enhance Fe tolerance in *A. sachalinensis*, facilitating the establishment of *A. sachalinensis* in soils containing high concentrations of heavy metals. Revegetation of old mine sites, including sedimentary sites, requires soil covering and afforestation after closing. Regarding this crucial issue, our study indicates that revegetation would be able to start at sedimentary sites in use, which may contribute to rapid afforestation. In the future, *A. sachalinensis* seedlings inoculated by *Ph. bamuru* will be planted in the study site and monitored to clarify the interaction between *A. sachalinensis* and *Ph. bamuru* in the field.

## Supporting information

S1 Fig Number and diameters at ground height of *A. sachalinensis* at a sedimentary site. **The number and diameter at ground height of** ****A. sachalinensis**** **were measured in August 2022**.
(TIF)

S2 Fig Colony of *Ph. bamuru* (BL211). ****Phialocephala bamuru**** **(BL211) was grown on 1% malt extract agar medium for 2 weeks at 23 °C in the dark. The scale bar represents 10 mm**.
(TIF)

S3 Fig Maximum likelihood phylogenetic tree of BL211 isolate based on a concatenated matrix composed of ITS region. **The tree with the highest log-likelihood was −1562.97. The percentages of trees in which the associated taxa clustered are shown next to the branches**.
(TIF)
