# Supplementary figures and images for: Abies sachalinensis naturally growing at a sedimentary site acquires iron tolerance via detoxicants production, elemental transfer adjustment, and root endophytic Phialocephala bamuru producing siderophores
Source: PLoS One. 2025 Jun 17;20(6):e0325294. doi: 10.1371/journal.pone.0325294 (PMC12173362; doi:10.1371/journal.pone.0325294)

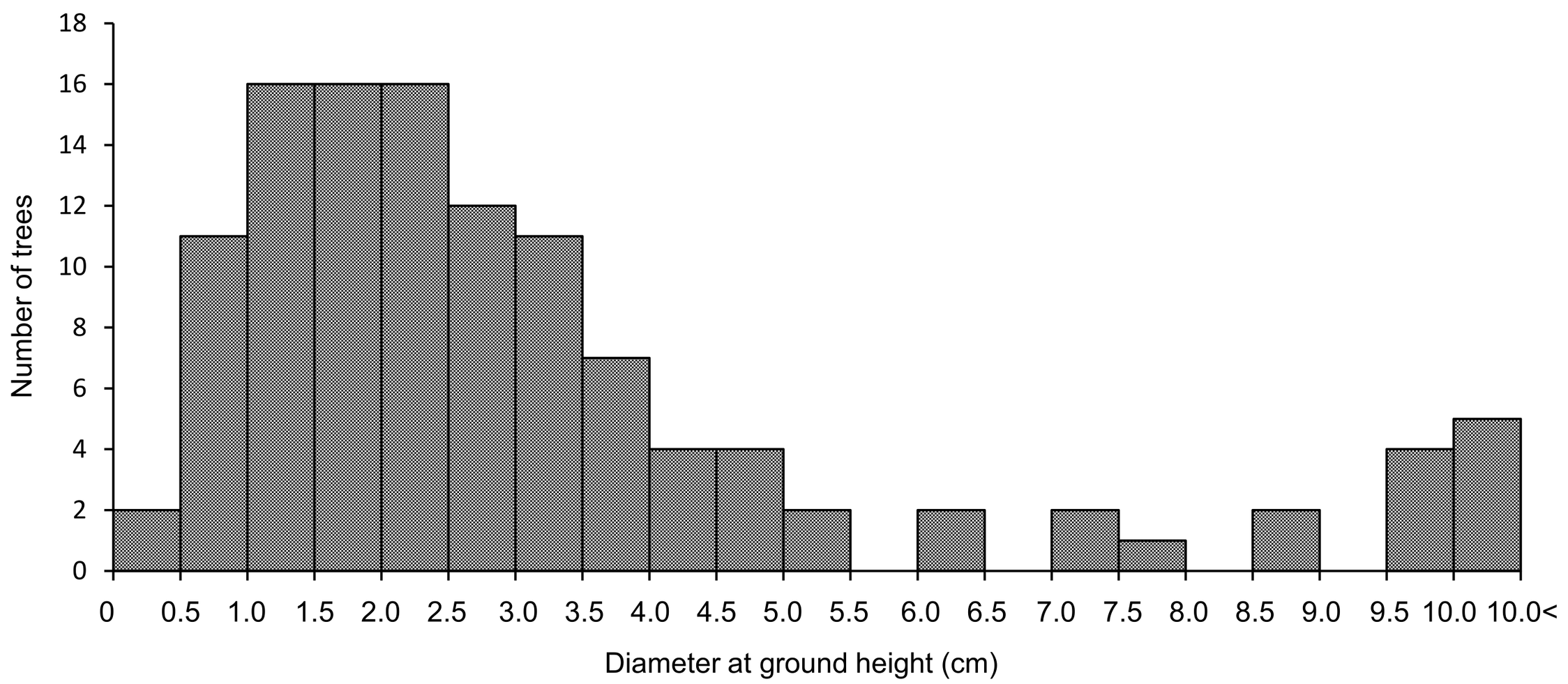

Supplement: S1 Fig — The number and diameter at ground height of A. sachalinensis were measured in August 2022. (TIF) [file pone.0325294.s001.tif]

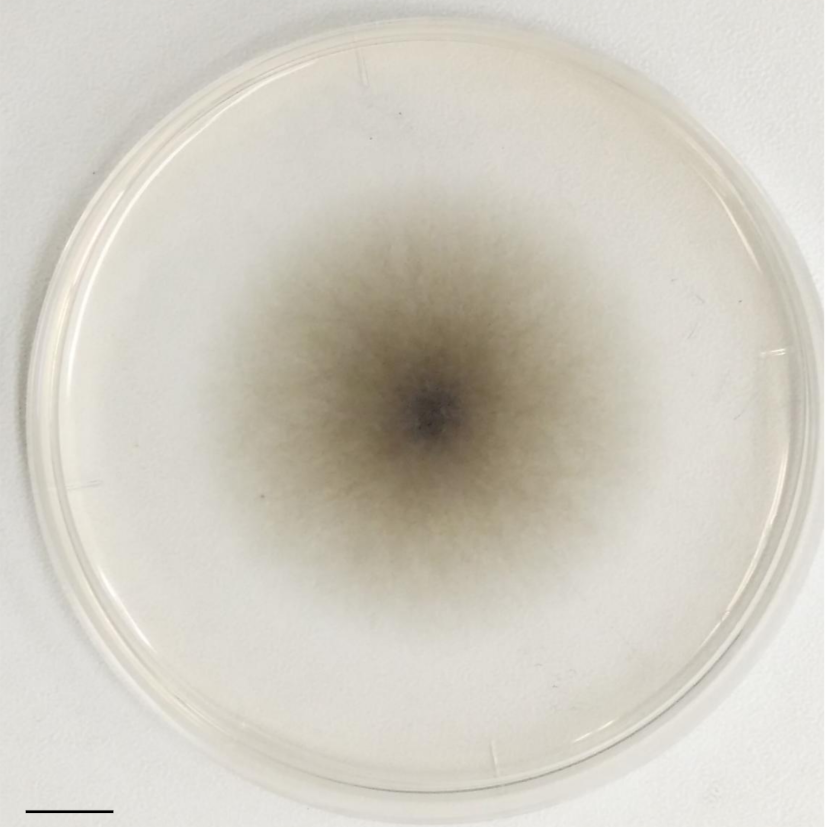

Supplement: S2 Fig — Phialocephala bamuru (BL211) was grown on 1% malt extract agar medium for 2 weeks at 23 °C in the dark. The scale bar represents 10 mm. (TIF) [file pone.0325294.s002.tif]

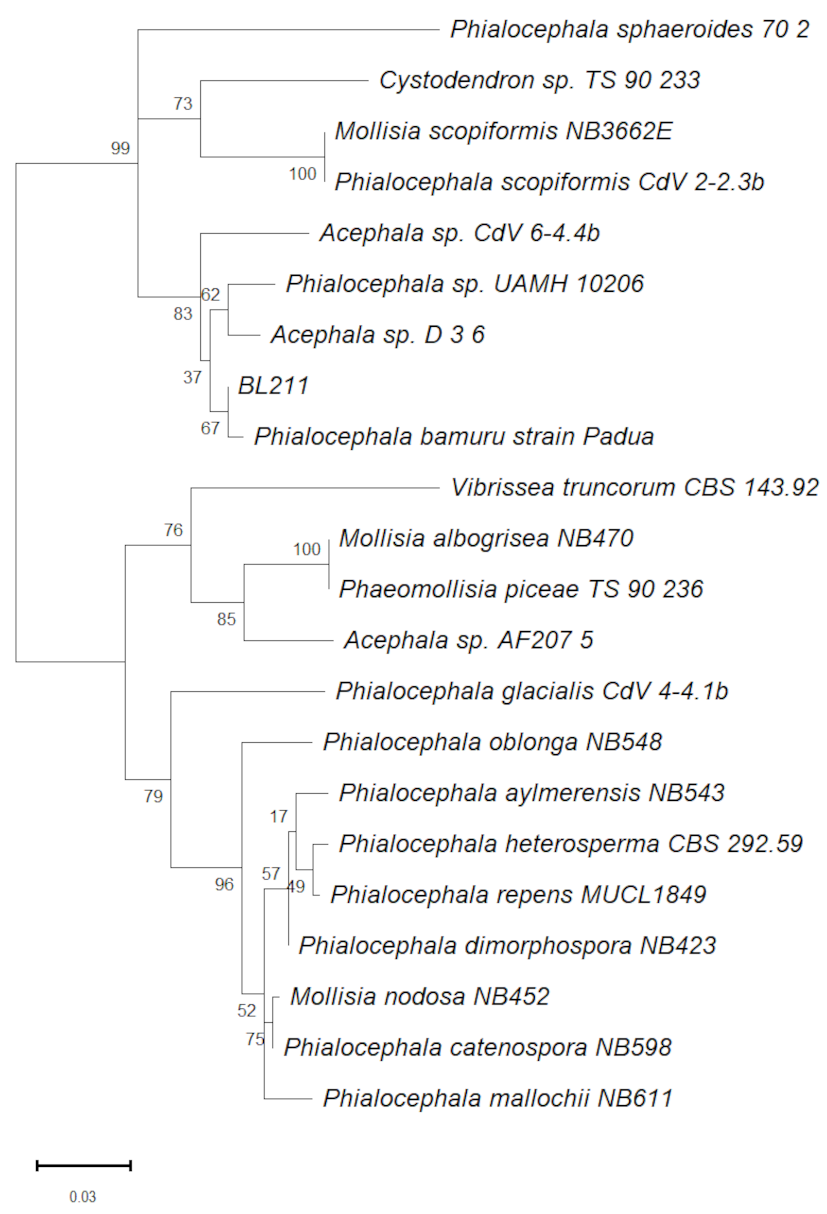

Supplement: S3 Fig — The tree with the highest log-likelihood was −1562.97. The percentages of trees in which the associated taxa clustered are shown next to the branches. (TIF) [file pone.0325294.s003.tif]
